# A comparative analysis of the complete chloroplast genomes of three *Chrysanthemum boreale* strains

**DOI:** 10.7717/peerj.9448

**Published:** 2020-07-03

**Authors:** Swati Tyagi, Jae-A Jung, Jung Sun Kim, So Youn Won

**Affiliations:** 1 Genomics Division, National Institute of Agricultural Sciences, Rural Development Administration, Jeonju, Republic of Korea; 2 Floriculture Research Division, National Institute of Horticultural and Herbal Science, Rural Development Administration, Wanju, Republic of Korea

**Keywords:** Asteraceae, Anthemideae, *Chrysanthemum*, Chloroplast genome, Phylogeny, Next generation sequencing

## Abstract

**Background:**

*Chrysanthemum boreale* Makino (Anthemideae, Asteraceae) is a plant of economic, ornamental and medicinal importance. We characterized and compared the chloroplast genomes of three *C. boreale* strains. These were collected from different geographic regions of Korea and varied in floral morphology.

**Methods:**

The chloroplast genomes were obtained by next-generation sequencing techniques, assembled de novo, annotated, and compared with one another. Phylogenetic analysis placed them within the Anthemideae tribe.

**Results:**

The sizes of the complete chloroplast genomes of the *C. boreale* strains were 151,012 bp (strain 121002), 151,098 bp (strain IT232531) and 151,010 bp (strain IT301358). Each genome contained 80 unique protein-coding genes, 4 rRNA genes and 29 tRNA genes. Comparative analyses revealed a high degree of conservation in the overall sequence, gene content, gene order and GC content among the strains. We identified 298 single nucleotide polymorphisms (SNPs) and 106 insertions/deletions (indels) in the chloroplast genomes. These variations were more abundant in non-coding regions than in coding regions. Long dispersed repeats and simple sequence repeats were present in both coding and noncoding regions, with greater frequency in the latter. Regardless of their location, these repeats can be used for molecular marker development. Phylogenetic analysis revealed the evolutionary relationship of the species in the Anthemideae tribe. The three complete chloroplast genomes will be valuable genetic resources for studying the population genetics and evolutionary relationships of Asteraceae species.

## Introduction

The genus *Chrysanthemum* belongs to the largest Angiosperm family, the Asteraceae (Hirakawa et al., 2019). *Chrysanthemum* species are economically important (Hirakawa et al., 2019). They are valued as cut flowers or potted garden flowers due to the diversity of their morphological traits including color, shape and size of the flower head, ray florets and disc florets (Shinoyama et al., 2012; Song et al., 2018). In addition, they are used as medicinal herbs in Korean and Chinese folk medicine (Won, Jung & Kim, 2018) for the treatment of inflammation, asthma and diarrhea, and as a traditional health food (Han et al., 2019; Sun et al., 2015; Wang et al., 2015). Polyploidy and hybridization events were reported to be responsible for evolution and speciation of *Chrysanthemum* genus (Liu et al., 2012; Ma et al., 2016; Yang et al., 2006), and *Chrysanthemum* species exhibit diverse ploidy levels (2*n* = 2*x* =18 to 2*n* = 10*x* = 90) (Chen et al., 2008). The commercial cultivar *Chrysanthemum* × *morifolium* Ramat. is a hexaploid species and its genetic studies on important traits and breedings are difficult.

*Chrysanthemum* includes around 40 different species native to Eurasia, especially in Korea, China and Japan (Liu et al., 2012). However, some species and varieties are narrowly distributed in specific habitats (Kondo et al., 2003; Liu et al., 2012). A total of 8 species, nine subspecies and one variety were reported in Korea (Hoang et al., 2020; Lee, 2006). Of particular importance to the present study is a wild relative, *Chrysanthemum boreale* Makino, which is a diploid species, bears small yellow flowers, and occurs in natural stands in eastern Asia (Hwang et al., 2013; Kim et al., 2014). Comparative transcriptomic analysis revealed that *C. boreale* diverged from *C. morifolium* about 1.7 million years ago (Won et al., 2017). *C. boreale* is resistant to one of the most destructive fungal diseases, namely white rust caused by *Puccinia horiana* Henn. (Park et al., 2014), and it has anti-inflammatory and skin-regenerative properties (Kim et al., 2015b, 2010). Several *C. boreale* strains collected from natural stands in Korea displayed variations in morphology such as leaf shapes and flower head, and in karyotype with the occurrence of aneuploidy (Hoang et al., 2020; Hwang et al., 2013). However, their genetic sequence divergence remains unknown. Currently, work is underway to sequence the nuclear genome of one *C. boreale* strain aiming to facilitate molecular, genetic, and physiological studies on *Chrysanthemum*. Molecular markers derived from both nuclear and chloroplast (cp) genomes would help reveal the relationships among strains and the genetic position of *C. boreale* in Asteraceae.

The cp genome encodes proteins that are key to photosynthesis and other metabolic processes (Liu et al., 2018b). The uni-parental inheritance of the cp genome (usually maternal in angiosperms and paternal in gymnosperms) and conserved gene content and order has made cp genome a valuable asset for plant phylogenetic and evolutionary studies (Birky, 2001; Wu & Ge, 2012). Plant cp genomes are generally between 120 kb and 160 kb in length and have a quadripartite circular structure comprising a pair of inverted repeat (IR) regions, a large single copy (LSC) region, and a small single copy (SSC) region (Thode & Lohmann, 2019). Advances in next-generation sequencing techniques have made it much easier to reconstruct the complete cp genome and uncover phylogenetic relationships at various taxonomic levels (Jansen et al., 2007; Moore et al., 2010; Parks, Cronn & Liston, 2009). Although the structure of cp genome is generally conserved, variation between species, subspecies, and individuals is present, and includes SNPs, indels, sequence rearrangements, IR expansion, gene loss and intron retention (Li et al., 2018). The cp genome sequences have helped to elucidate the phylogenetic relationships and evolutionary history of many plant species, including rice (*Oryza* AA genome), vegetables in the *Brassica* genus, and conifer tree (*Pinus taeda* L.) (Asaf et al., 2018; Kim et al., 2018, 2015a).

Here, we analyzed the cp genomes of three morphologically different *C. boreale* strains collected from different geographic regions in Korea. We discovered their phylogenetic relationships to other species in the tribe Anthemideae, including *Chrysanthemum* species. This study provides useful genomic information for molecular evolutionary and phylogenetic studies of Asteraceae, and genetic resources for breeding and improvement of chrysanthemum.

## Materials and Methods

### Ethics statement

The plant sample used in this study is neither endangered nor protected, and was collected from an area that was not privately owned or protected in any way. No specific permits were required to conduct this study.

### Plant materials and sequencing

Two *C. boreale* strains with morphological differences were collected from different locations (Fig. S1) in the Republic of Korea and deposited at the National Agrobiodiversity Center, Rural Development Administration. The strain from Gongju-si, Chungcheongnam-do was labeled IT232531, and the one from Suwon-si, Gyeonggi-do was labeled IT301358. The total DNA was isolated from fresh leaves as previously described (Kim et al., 2006). The quality and quantity of DNA were examined using a Nanodrop 2000 spectrophotometer (Thermo Fisher Scientific, Waltham, MA, USA) and gel electrophoresis (in 0.8% agarose). Paired-end libraries of 350-bp insert size were constructed using TruSeq DNA PCR-Free kit (Illumina, San Diego, CA, USA) and sequenced with a 101-bp read length by Macrogen (Republic of Korea) using the HiSeq4000 (Illumina, San Diego, CA, USA) according to the manufacturer’s instructions. Another *C. boreale* strain, labeled 121002, was collected from Jeongeup-si, Jeollabuk-do (Hwang et al., 2013) and its cp genome was sequenced. Our group had previously submitted this cp genome sequence to NCBI with accession number MG913594 (Won, Jung & Kim, 2018).

### Chloroplast genome assembly and annotation

The complete cp genome was assembled de novo (Kim et al., 2015a). Briefly, raw reads were trimmed using the Trimmomatic program (Bolger, Lohse & Usadel, 2014), assembled using the clc_assembler in the CLC Genomics Workbench v6.0 (CLC Bio, Denmark, Europe). Gaps were filled using Gap Closer (Luo et al., 2012). The resulting contigs were searched for cp-encoding contigs by BLASTN analysis against the cp genome of *C. boreale* strain 121002, and circularized. These were annotated using the online programs Dual Organellar GenoMe Annotator, cpGAVAS v.2.0 and BLAST (Shi et al., 2019; Wyman, Jansen & Boore, 2004). The structure of transfer RNA (tRNA) was predicted using the tRNAscan-SE 1.21 program using the default settings (Schattner, Brooks & Lowe, 2005). The circular genome map with structural features was generated using the OGDRAW v1.2 program (Lohse et al., 2013). The resulting cp genome sequences of strains IT232531 and IT301358 were deposited in NCBI under the IDs MN909052 and MN913565, respectively.

### Chloroplast genome comparison

The cp genomes of the three *C. boreale* strains were compared using the mVISTA program in the Shuffle-LAGAN mode, using the annotation of strain 121002 as the reference (Frazer et al., 2004). The SNPs and indels in the cp genome were also recorded using DnaSP6.0 (Rozas et al., 2017) and manually verified from the sequence alignment by Clustal Omega (Sievers et al., 2011).

### Characterization of repetitive sequences

Simple sequence repeats (SSRs) were discovered using the online web tool MISA (http://pgrc.ipk-gatersleben.de/misa/) with the following parameters: ten repetitions for mononucleotide motifs, eight for dinucleotide motifs, four for tri- and tetra-nucleotide motifs, and three for penta-and hexa-nucleotide motifs (Beier et al., 2017). Next, four different types of repeats, namely forward (F), palindromic (P), reverse (R) and complement (C) repeats were analyzed using the REPuter program (https://bibiserv.cebitec.uni-bielefeld.de/reputer) with a minimum repeat size of 30 bp and a Hamming distance of 3 (Kurtz et al., 2001). To reduce redundancy, IRb sequence was removed before analysis and repeats detected at the same position were merged into single repeat.

### Phylogenetic analysis

The entire cp genomes and 77 protein-coding sequences shared in cp genomes of species belonging to tribe Anthemideae were used to reconstruct the phylogenetic relationships. *Lactuca sativa* L. was used as the outgroup. The species and the accession numbers of their cp genomes in NCBI are listed in Table S1. The nucleotide sequences were aligned using Clustal Omega (Sievers et al., 2011). Maximum likelihood (ML) analyses were conducted using the IQ-TREE web server (http://iqtree.cibiv.univie.ac.at) with the best-fit models determined by ModelFinder in the IQ-TREE package (Table S2) and 1,000 bootstrap replicates (Hoang et al., 2018; Kalyaanamoorthy et al., 2017; Nguyen et al., 2015). Bayesian inferences (BI) were performed with MrBayes v. 3.2.7 (Ronquist et al., 2012) and the nucleotide substitution models determined by ModelTest-NG (Darriba et al., 2019) (Table S2). The Markov chain Monte Carlo algorithms were run for 10 million generations and sampled every 1,000 generations. The first 25% of trees were discarded as burn-in and the remaining trees were used to build a majority-rule consensus tree with posterior probability values for each node. The stationary was considered to be reached when the average standard deviation of split frequencies remained below 0.01. The phylogenetic tree was visualized with FigTree v1.4.4 (http://tree.bio.ed.ac.uk/software/figtree/).

## Results

### Characterization of chloroplast genomes

**Figure 1:**
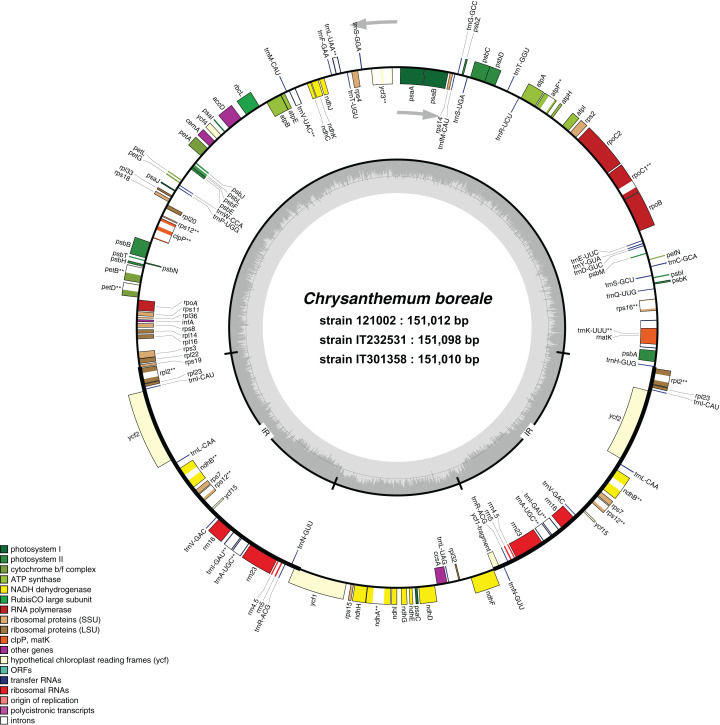
Genome map of *Chrysanthemum boreale* chloroplast genomes. Thick lines indicate the extent of the inverted repeat regions, which separate the genome into large and small single copy regions. Genes drawn inside the circle are transcribed clockwise, while those outside of the circle are transcribed counter clockwise. Genes belonging to different functional groups are color coded differently. The dark gray in the inner circle corresponds to the GC content while the light gray corresponds to the AT content. Genes with introns are marked with an asterisk.

We used NGS techniques to generate approximately 30.2 Gb and 34.9 Gb of raw reads from strains IT232531 and IT301358, respectively. We assembled de novo the complete cp genomes of sizes 151,098 bp for IT232531 and 151,010 bp for IT301358 (Table 1). For comparison, we included the previously reported cp genome of *C. boreale* strain 121002, which was 151,012 bp in size (Won, Jung & Kim, 2018). All the three *C. boreale* strains had a typical quadripartite structure of cp genomes with an LSC, an SSC, and a pair of IR regions (Fig. 1). The length of the LSC region was 82,817 bp, 82,880 bp and 82,788 bp for the strains 121002, IT232531 and IT301358, respectively. The SSC region measured 18,281 bp, 18,312 bp and 18,310 bp in the strains 121002, IT232531 and IT301358, respectively. The strains were comparable in terms of the length of the IR regions and the GC content of the LSC, SSC, IR regions and the complete genome (Table 1). The IR regions had a higher GC content than the LSC and SSC regions due to the presence of GC-rich ribosomal RNA (rRNA) genes and tRNA genes in these regions.

**Table 1:** Summary of complete chloroplast genomes of three *Chrysanthemum boreale* strains.

Attributes	121002	IT232531	IT301358
Total size (bp)	151,012	151,098	151,010
LSC size (bp)	82,817	82,880	82,788
SSC size (bp)	18,281	18,312	18,310
IR size (bp)	24,957	24,953	24,956
Total GC content (%)	37.5	37.5	37.5
LSC GC content (%)	35.6	35.5	35.6
SSC GC content (%)	30.9	30.8	30.9
IR GC content (%)	43.1	43.1	43.1
Number of unique genes	113	113	113
Number of unique protein-coding genes	80	80	80
Number of unique tRNA genes	29	29	29
Number of unique rRNA genes	4	4	4
Genes duplicated	17	17	17
Genes with intron	16	16	16
Pseudogene	1	1	1

The cp genomes of all the strains comprised 113 unique genes. These included 80 protein-coding genes, 29 tRNA genes and four rRNA genes (Table 2). Each strain contained 61 protein-coding genes and 21 tRNA genes in the LSC region and 11 protein-coding genes and one tRNA gene in the SSC region (Fig. 1). Three genes (*rps12*, *rps19* and *ycf1*) were distributed in both single copy and IR regions. The IR regions contained seven protein-coding genes, seven tRNA genes and four rRNA genes each. Because the IR regions are duplicates of each other, all genes in these regions were also duplicated.

**Table 2:** List of genes in the *C. boreale* chloroplast genomes.

Category	Group of genes	Name of genes
Self-replication	Large subunit of ribosomal proteins	rpl2*(2x), *14, 16, 20, 22, 23*(2x), *32, 33, 36*
	Small subunit of ribosomal proteins	*rps2, 3, 4, 7*(2x), 8, 11, 12**(2x), 14, 15, 16*, 18, 19
	DNA dependent RNA polymerase	rpoA, B, C1*, C2
	rRNA genes	*rrn16*(2x), *rrn23*(2x), *rrn4.5*(2x), *rrn5*(2x)
	tRNA genes	trnA-UGC*(2x), *trnC-GCA, trnD-GUC, trnE-UUC, trnF-GAA, trnfM-CAU, trnG-GCC, trnH-GUG, trnI-CAU*(2x), trnI-GAU*(2x), trnK-UUU*, trnL-CAA(2x), trnL-UAA*, trnL-UAG, trnM-CAU, trnN-GUU(2x), *trnP-UGG, trnQ-UUG, trnR-ACG*(2x), *trnR-UCU, trnS-GCU, trnS-GGA, trnS-UGA, trnT-GGU, trnT-UGU, trnV-GAC*(2x), trnV-UAC*, trnW-CCA, trnY-GUA
Photosynthesis	Photosystem I	*psaA, B, C, I, J*
	Photosystem II	*psbA, B, C, D, E, F, H, I, J, K, L, M, N, T, Z*
	NADH dehydrogenase	ndhA*, B*(2x), *C, D, E, F, G, H, I, J, K*
	Cytochrome b6/f complex	petA, B*, D*, G, L, N
	ATP synthase	atpA, B, E, F*, H, I
	Rubisco	*rbcL*
Other genes	Translational initiation factor	*infA*
	Maturase	*matK*
	Protease	clpP*
	Envelop membrane protein	*cemA*
	Subunit Acetyl-CoA-Carboxylase	*accD*
	C type cytochrome synthesis gene	*ccsA*
Unknown	Conserved open reading frame	*ycf1*, *2*(2x), 3*, 4, 15(2x)

**Notes:**

* Intron-containing genes.

** Trans-spliced gene.

The duplicated genes are shown with (2x) next to the gene name.

The cp genomes of *C. boreale* included 16 intron-containing genes (Table 3). The genes *ycf3* and *clpP* had two introns each, while all other genes contained a single intron. Nine of the introns were identical in length, whereas seven other introns differed in length between 1 bp and 24 bp. The intron of the *trnK-UUU* gene was largest (2,560–2,575 bp) in all the strains and its pairwise length differed between the strains by 7–15 bp. The intron of the *ndhA* gene in IT232531 was 24 bp and 6 bp longer than that in strains 121002 and IT301358, respectively. In each strain, the *rps12* gene was trans-spliced, with the 5′ end exon located in the LSC region and the duplicated 3′ end exon located in both the IR regions, as previously reported in other plants (Thode & Lohmann, 2019).

**Table 3:** Comparison of introns length of *C. boreale* strains in cp genome.

No.	Genes	Location		121002	IT232531	IT301358
1	*atpF*	LSC		699	699	699
2	*clpP*	LSC	Intron1	608	609	611
			Intron2	800	797	797
3	*ndhA*	SSC		1045	1069	1063
4	*ndhB*	IR		670	670	670
5	*petB*	LSC		747	746	747
6	*petD*	LSC		675	675	675
7	*rpl2*	LSC		662	662	662
8	*rpoC1*	LSC		732	732	732
9	*rps12*	IR		535	535	535
10	*rps16*	LSC		881	892	887
11	*ycf3*	LSC	Intron1	740	743	743
			Intron2	711	713	711
12	*trnA-UGC*	IR		812	812	812
13	*trnI-GAU*	IR		776	776	776
14	*trnK-UUU*	LSC		2568	2575	2560
15	*trnL-UAA*	LSC		424	423	425
16	*trnV-UAC*	LSC		572	572	572

Given that the cp genome of *C. boreale* strain 121002 was obtained using PacBio’s long reads (Won, Jung & Kim, 2018), we repeated the cp genome assembly of 121002 using Illumina’s short reads as conducted for other *C. boreale* strains. The sequence comparison between two cp genomes obtained using long reads and short reads revealed that there was no SNP detected. Instead, indels were observed at four genomic regions and all of them were associated with homopolymers. Three indels were located in intergenic spacers (IGSs), *trnE-UUC_rpoB* (18 thymines in the long-read assemble vs. 17 thymines in the short-read assemble) and *psaA_ycf3* (16 vs. 15 adenines), and the intron of *rpl16* (8 vs. 9 cytosines), which didn’t change the protein sequences. However, the coding region of *ycf1* possessed one indel (13 adenines vs. 14 adenines) (Fig. S2), which resulted in 1,036 amino acids (aa) in the original data due to the premature stop codon. However, one-bp insertion generated the *ycf1* protein of 1,668 aa, which was more consistent with the other *C. boreale* strains (1,672 aa in IT232531 and 1,673 aa in IT301358). While we used the original cp sequence deposited in NCBI for analyses, in case of *ycf1*, we used the newly obtained sequences.

### Variation in chloroplast genomes

**Figure 2:**
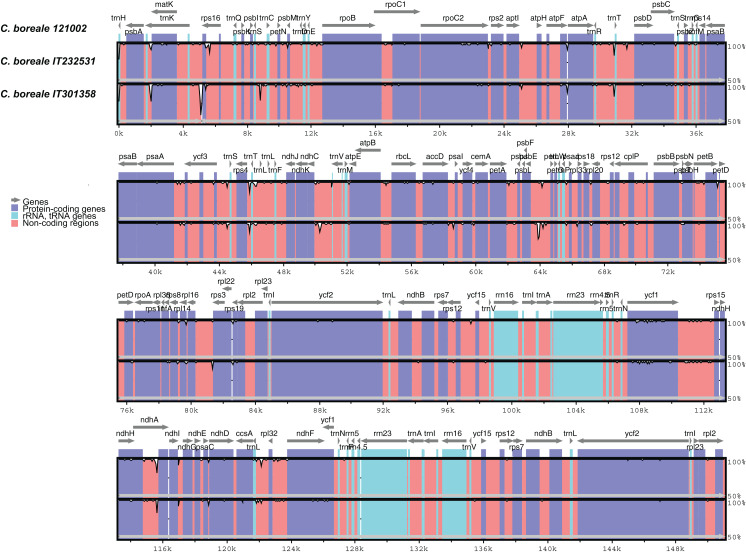
Comparison of chloroplast genomes of *C. boreale* strains using the mVISTA program. A cut-off of 70% identity was used for the plots. The *Y*-scale axis represents the percent identity between 50% and 100%.

The mVISTA-based identity plot indicated conservation in DNA sequence and gene synteny across the whole cp genome, and revealed the regions with increased genetic variation (Fig. 2). The gene number, order and orientation were conserved. There was higher genetic variability in the single copy (LSC and SSC) regions than in the IR regions, and in non-coding regions than in coding regions. Highly diverged regions included the IGSs, *trnK-UUU_rps16*, *trnS-GCU_trnC-GCA*, *trnR-UCU_trnT-GGU*, *rps4_trnL-UAA*, *ndhC_trnV-UAC*, *psbE_petL*, *rps16_rps3*, and *trnL-UAG_rpl32* and the introns of *trnK-UUU*, *rps16* and *ndhA* (Fig. 2). We detected a total of 298 SNPs (Table S3). The LSC region contained a majority of the SNPs (204, accounting for 68.5% of the SNPs), followed by the SSC region (75, 25.2%), and the IR regions (19, 6.4%). The SNPs were more abundant in non-coding regions: 141 were located in intergenic regions, 46 in introns, and 111 in coding regions. The *ycf1* gene contained the largest number of substitutions (25 SNPs), followed by the *trnK-UUU* intron (18 SNPs), *rpoC2* (12 SNPs) and the *ycf1_rps15* IGS (11 SNPs).

We detected a total of 106 indels (Table S4): 81 in the LSC, 19 in the SSC, and six in the IR regions. A total of 86 and 17 indels were located in IGS and introns, respectively, whereas three were contained in coding regions. The *ndhC_trnV-UAC* spacer had five indels, while the introns of *trnK-UUU* and *ndhA*, and the spacers of *psaA_ycf3* and *psbE_petL* contained four indels each. The *psbE_petL* IGS included the two largest indels (54 bp and 36 bp) in the cp genome. The *trnK-UUU* intron was the longest in the genome, and one of the most variable regions, comprising both SNPs and indels (Fig. S3). The 5-bp deletion at the end of the protein-coding gene *rpoC2* in strain IT232531 generated a protein that was longer by two amino-acids. In the *ycf1* gene, the 3-bp insertion in strain IT301358 did not change the protein’s translational frame.

**Figure 3:**
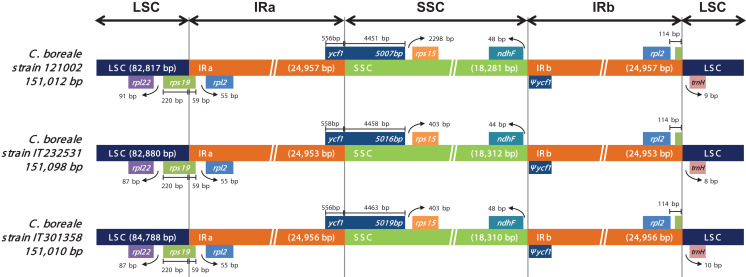
Comparison of the LSC, IR and SSC junction positions in the chloroplast genomes of the *C. boreale* strains. Genes above the longer box are transcribed in forward direction and genes below the box are transcribed in reverse direction. Ψ indicates a pseudogene.

We investigated the position of genes at the junction regions (LSC/IRa, IRa/SSC, SSC/IRb and IRb/LSC; Fig. 3). At the LSC/IRa junction, *C. boreale* possessed *rps19* with 220 bp in LSC and 59 bp in IRa. The IRa/SSC junction contained the functional *ycf1*, while the SSC/IRb possessed the duplicated partial copy, pseudogene *ycf1* (Ψ*ycf1*) and *ndhF*. At the IRb/LSC junction, *rpl2* and *trnH-GUG* were located within the distance of 122–124 bp from each other.

### Repeat analysis

**Figure 4:**
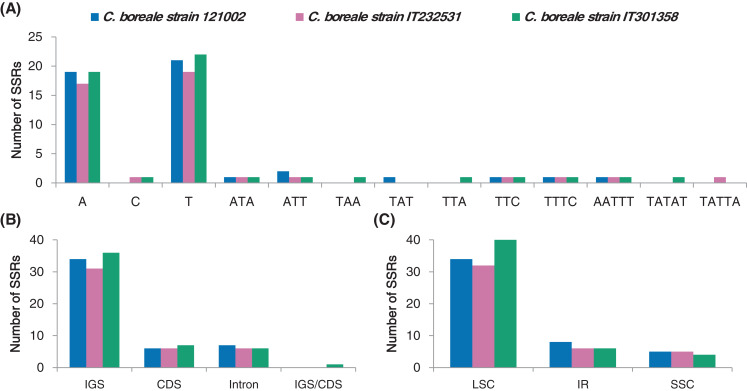
Analyses of simple sequence repeats (SSRs) in *C. boreale* chloroplast genomes. (A) The frequency of SSRs per sequence type. (B) The frequency of SSRs in intergenic spacer (IGS), coding sequence (CDS), intron and IGS/CDS. IGS/CDS represents SSRs shared in IGS and CDS. (C) The frequency of SSRs in large single copy (LSC), inverted repeat (IR) and small single copy (SSC) regions.

We investigated the distribution of SSRs that were 1–6 bp in length in the *C. boreale* cp genomes. We recorded a total of 47, 43 and 50 SSR motifs in 121002, IT232531 and IT301358, respectively (Table S5). Mononucleotide repetition was most prevalent in each cp genome, followed by tri-, penta-and tetra-nucleotide repetition (Fig. 4A). We did not detect di-or hexa-nucleotide SSRs. In terms of sequence context, there were more adenine and thymine residues than cytosine and guanine residues (Fig. 4A). Intergenic and intronic regions contained more SSRs than coding regions, with 41, 37 and 42 instances of SSR occurrence in the non-coding regions in the 121002, IT232531 and IT301358 strains, respectively (Fig. 4B). Most of the SSRs were located in the LSC region followed by those in the IR region (Fig. 4C).

**Figure 5:**
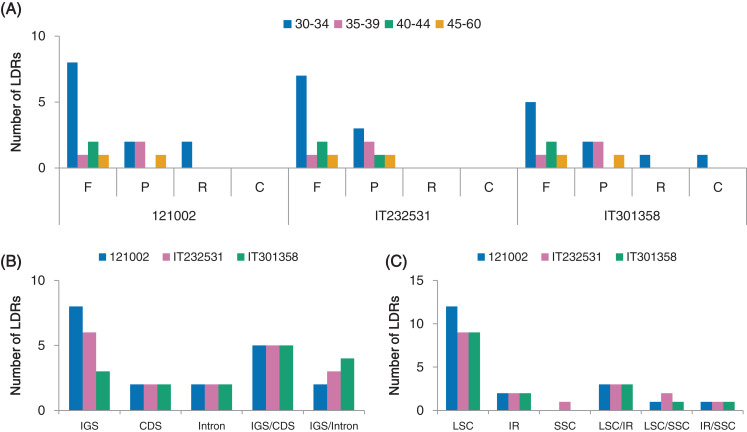
Analyses of long dispersed repeats (LDRs) in *C. boreale* chloroplast genomes. (A) The frequency of LDRs classified by the length and type of repeat: forward (F), palindromic (P), reverse (R) and complement (C) repeats. (B) The frequency of LDRs in intergenic spacer (IGS), coding sequence (CDS), intron, IGS/CDS and IGS/intron. IGS/CDS represents LDRs shared in IGS and CDS. IGS/intron represents LDRs shared in IGS and intron. (C) The frequency of LDRs in different genomic regions.

We detected four different types of long dispersed repeats (LDRs), namely forward (F), palindromic (P), reverse (R) and complement (C) repeats, each with a motif length longer than 30 bp. We identified a total of 19 (12F, 5P, 2R), 18 (11F, 7P) and 16 (9F, 5P, 1R, 1C) repeats in the cp genomes of strains 121002, IT232531 and IT301358, respectively (Fig. 5A; Table S6). F and P repeats were more abundant than C and R repeats. Repeat units of 30–34 bp were the most common, whereas repeat units longer than 40 bp occurred less frequently (Fig. 5A). More LDRs were located in non-coding regions (IGS and introns) than in coding regions (Fig. 5B). Among the protein-coding genes, LDRs were detected in the *psaA*, *psaB* and *ycf2* in all three *C. boreale* strains (Table S6). Most LDRs were present in LSC region compared to IR and SSC regions, while some LDRs were shared among LSC, IR and SSC regions (Fig. 5C).

### Phylogenetic analysis

**Figure 6:**
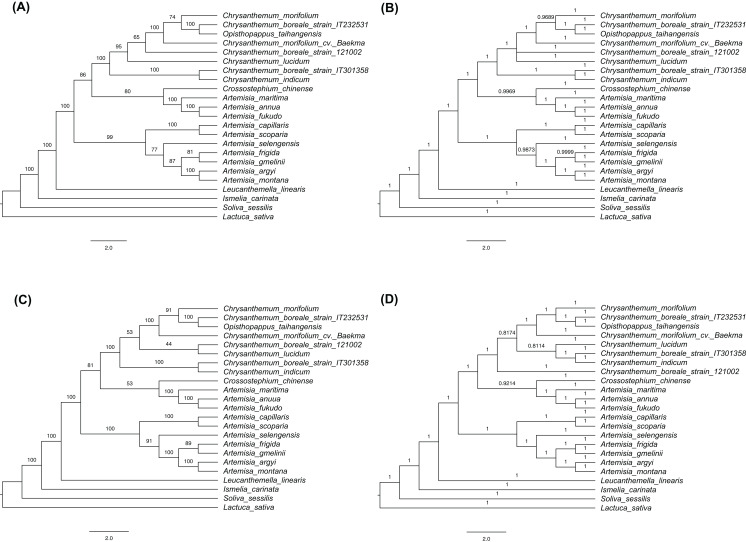
Cladograms using the maximum likelihood (ML) and Bayesian inference (BI) methods. (A) ML tree based on the sequences of 77 shared protein-coding genes. (B) BI tree based on the sequences of 77 shared protein-coding genes. (C) ML tree based on the complete chloroplast genomes. (D) BI tree based on the complete chloroplast genomes. Numbers above the branches indicate bootstrap support values in ML trees and BI posterior probability in BI trees.

The phylogenetic trees were constructed based on complete cp genome sequences and 77 protein-coding genes that were common to the three *C. boreale* strains, the 17 other species of the tribe Anthemideae (Asteroideae, Astereaceae), and the outgroup species, *L. sativa* (Cichorieae, Cichorioideae, Asteraceae). The multiple alignment of complete cp genomes contained 158,397 nucleotide sites in which 11,844 were variable and 3,371 were parsimony informative. The multiple alignment of protein-coding sequences possessed 62,965 nucleotide sites in which 3,065 were variable and 900 were parsimony informative. In each cp sequences, both ML and BI trees revealed similar topologies but minor difference within *Chrysanthemum* species (Fig. 6; Fig. S4). Two datasets also resulted in similar phylogenetic relationship. All *Chrysanthemum* sequences were grouped into a single clade together with *Opisthopappus taihangensis* (Ling) C.Shih with high bootstrap support and Bayesian inference (Fig. 6). Three *C. boreale* strains were all non-monophyletic, which was also observed in two *C. morifolium* analyzed. Additionally, *Artemisia* species were clustered into two clades. Among them, seven species formed a monophyletic group, and other three were located in another clade and were closer to the *Chrysanthemum* clade.

## Discussion

The overall cp genome structures and sequences in the three *C. boreale* strains examined here were conserved and displayed the classical quadripartite structure of land plant cp genomes (Shen et al., 2018). The gene content, gene order and gene orientation in the cp genomes were conserved. Genomic rearrangements such as inversion of the SSC or of individual genes is common in cp genomes (Liu et al., 2018a). However, there were no definitive genomic rearrangements or gene inversions in the three *C. boreale* strains. The length differences of cp genomes were observed among strains, which was due to variation mainly in the lengths of the LSC and SSC regions. The IR region, which influences the evolution of cp genomes due to its expansion, contraction, or complete loss (Wicke et al., 2011; Zhu et al., 2016), was similar in length, with only a 1–4 bp difference among strains. Our results are consistent with similar studies of the cotton genus (*Gossypium*), in which the length of LSC regions accounted for the cp genome size difference (Chen et al., 2017). This is different from studies in duckweed species (Lemnoideae), in which differences in cp genome size were due to differences in IR regions (Ding et al., 2017).

Sequence identity plot and the analyses of SNPs and indels revealed the variable regions in the cp genome of *C. boreale*. In line with observations in other plant species, the LSC and SSC regions were more divergent than the IR regions, and non-coding regions were more variable than coding regions (Meng et al., 2019; Wang et al., 2018). Among the variable regions in the *C. boreale* cp genome, the introns of *trnK-UUU* and *ndhA*, and the spacers of *ndhC_trnV-UAC*, *ycf1_rps15*, *trnL-UAG_rpl32*, and *psbE_petL* as well as the coding regions of *ycf1* and *rpoC2* contained many polymorphisms, suggesting rapid genome evolution due to higher mutation rates than other regions. The *trnK-UUU* intron was longer than 2.5 kb and encompassed *matK*, which included six SNPs. This region has been extensively used as a molecular marker for phylogenetic and evolutionary studies (Hausner et al., 2006). Therefore, future studies investigating phylogeny and evolution in relatives of *C. boreale* are likely to find its cp genome a useful resource.

We also detected variation in the number and distribution of two types of repeats, SSRs and LDRs, in both non-coding (IGS and intron) and coding regions. The occurrence of repeats was more prevalent in the non-coding regions than in the coding regions, similar to reports in other species (Kim et al., 2015a; Meng et al., 2019; Shen et al., 2018). Differential distribution of these repeats is associated with cp genome rearrangement and nucleotide substitution (Weng et al., 2014). Therefore, these repeats could be used to develop genetic markers for phylogenetic studies. The obtained SSR repeats, together with the variable regions could be used to examine the genetic structure, diversity, phylogeny, and differentiation of *Chrysanthemum* and other Asteraceae species.

The phylogenetic analysis revealed the evolutionary relationships of species in the Anthemideae tribe. The investigated species were clustered into a monophyletic group and were largely classified into two groups: *Chrysanthemum* and *Artemisia*. The *Chrysanthemum* clade included *Chrysanthemum* species, *O*. *taihangensis, Crossostephium chinense* Makino, and unexpected three *Artemisia* species (*A. annua, A. fukudo* and *A.maritima*), while the *Artemisia* clade included the remaining seven *Artemisia* species, which was consistent with the previous analysis (Gu et al., 2019). However, other phylogenetic studies showed that all *Artemisia* species were clustered together and separated from its sister genus *Chrysanthemum* (Meng et al., 2019; Shahzadi et al., 2020). In their analyses, the three *Artemisia* species closer to *Chrysanthemum* in our study formed a separated clade within the *Artemisia* genus. At least, it is clear that *Artemisia* species are classified into two groups based on cp sequences but their relationship with *Chrysanthemum* needs to be further addressed.

Within the *Chrysanthemum* clade, *C. boreale* strains were placed in separate branches. Two *C*. *morifolium* cultivars from Korea and China were also placed in separate branches. This was similar to an earlier phylogenetic analysis of more diverse *Chrysanthemum* species that used seven cp regions and a single copy nuclear gene (the *chrysanthemyl diphosphate synthase*, *CDS* gene): different strains of *C. indicum* were located in different branches (Liu et al., 2012). On the other hand, we assembled the nuclear genomic regions encompassing the rRNAs and the nuclear ribosomal internal transcribed spacer (nrITS) of around 5.8 kb in size for three *C. boreale* strains and *C. morifolium* cv. *Baekma* with the same approach for cp genome (Supplemental Data 1). Their phylogenetic relationships based on nuclear sequences indicated that all *Chrysanthemum* sequences formed a monophyletic group in which *C. boreale* strain IT301358 was clustered together with *C. morifolium* cv. *Baekma* (Fig. S5). These results suggest the close affinity within the *Chrysanthemum* genus and therefore the classification or circumscription using cp and nrITS sequences would be difficult within *Chrysanthemum*. Divergence and speciation in the *Chrysanthemum* genus were suggested to be affected by geographical and ecological factors (Liu et al., 2012). Further research including other cultivars and varieties from different regions, and molecular markers from nucleus genome sequences, may reveal the origin of cultivated chrysanthemum and the genetic relationships within the *Chrysanthemum* group.

*Opisthopappus taihangensis* is a monotypic species in the genus (Gu et al., 2019) and its phylogenetic position as a sister taxon of *C. boreale* was inconsistent with previous studies in which *O. taihangensis* was basal to the *Chrysanthemum* group when nrITS sequences were used (Zhao et al., 2010). Considering that nrITS sequences can be as short as 447 bp (Zhao et al., 2010), we would expect fewer informative polymorphisms from ITS than the cp as a whole. However, we cannot exclude the possibility that the phylogeny based on the cp genome was sometimes unreliable due to the mode of inheritance of cp genome (Folk, Mandel & Freudenstein, 2017; Tonti-Filippini et al., 2017). Hybridization between distant species (or relatives) and the subsequent chloroplast capture have also been suggested to underlie discrepancies between the nuclear and cp genomes and consequently cause differences in phylogenetic analysis. Phylogenetic trees based on cp and nuclear data also showed the incongruence within *Chrysanthemum* as discussed above.

## Conclusions

Using next-generation sequencing technology, we compared the complete cp genomes of three *C. boreale* strains. The gene content, gene order and GC content of all the three cp genomes were conserved. The rapidly evolving divergent regions and repeats we identified could potentially serve as molecular markers in phylogenetic studies. Phylogenetic analyses using other *Chrysanthemum* species and other species within Anthemideae strongly supported the taxonomic status of the strains within the tribe. The data presented here provide insights into the evolutionary relationships among *C. boreale* strains and other *Chrysanthemum* species, and will act as a valuable resource for their molecular identification and breeding, as well as for further biological discoveries.

## Supplemental Information

10.7717/peerj.9448/supp-1 Supplemental Information 1 Characteristics of three *Chrysanthemum boreale* strains. (A) The collection areas are marked as “a” for 121002, “b” for IT232531 and “c” for IT301358. (B) The morphology of flower head, ray floret and leaf. The ruler scale is in mm.

10.7717/peerj.9448/supp-2 Supplemental Information 2 Alignment of nucleotide sequences of the *ycf1* gene. Only the inconsistent regions between two assembly processes are shown. The number in bp on the left indicates the position of first nucleotide displayed in the coding sequences of *ycf1*.

10.7717/peerj.9448/supp-3 Supplemental Information 3 Alignment of nucleotide sequences at the intron of *trnK-UUU*. Only the most divergent regions are shown. The number in bp on the left indicates the position of nucleotide in the complete chloroplast genome.

10.7717/peerj.9448/supp-4 Supplemental Information 4 Phylogenetic analyses using the maximum likelihood (ML) and Bayesian inference (BI) methods. (A) ML tree based on the sequences of 77 shared protein-coding genes. (B) BI tree based on the sequences of 77 shared protein-coding genes. (C) ML tree based on the complete chloroplast genomes. (D) BI tree based on the complete chloroplast genomes. Numbers above the branches indicate bootstrap support values in ML trees and BI posterior probability in BI trees. The scale bars indicate the number of nucleotide substitutions per site.

10.7717/peerj.9448/supp-5 Supplemental Information 5 Maximum likelihood tree based on nrITS sequences. Numbers above the branches indicate bootstrap support values. The scale bars indicate the number of nucleotide substitutions per site.

10.7717/peerj.9448/supp-6 Supplemental Information 6 List of chloroplast genomes used for phylogenetic analysis in this study.

10.7717/peerj.9448/supp-7 Supplemental Information 7 The best-fit models for phylogenetic analyses of different chloroplast datasets.

10.7717/peerj.9448/supp-8 Supplemental Information 8 Distribution of SNPs in the chloroplast genome of *C. boreale*.

10.7717/peerj.9448/supp-9 Supplemental Information 9 Distribution of indels in the chloroplast genome of *C. boreale*..

10.7717/peerj.9448/supp-10 Supplemental Information 10 Distribution of SSRs in the chloroplast genome of *C. boreale*.

10.7717/peerj.9448/supp-11 Supplemental Information 11 Distribution of long dispersed repeats in the chloroplast genome of *C. boreale*.

10.7717/peerj.9448/supp-12 Supplemental Information 12 The nucleotide sequences of the ribosomal RNAs and the nuclear ribosomal internal transcribed spacer. Sequences from three *C. boreale* strains and *C. morifolium* cv. Baekma were provided.

## Additional Information and Declarations

### DNA Deposition

The following information was supplied regarding the deposition of DNA sequences: The chloroplast genome sequences of *Chrysanthemum boreale* are available at GenBank: MN909052 (strain IT232531) and MN913565 (strain IT301358).

### Data Availability

The following information was supplied regarding data availability: Additional data are available in the Supplemental Figures and Tables, and the nucleotide sequences of nrITS regions in three *C. boreale* strains and *C. morifolium* cv. *Baekma* are available in a Supplemental File.
