# Supplementary figures and images for: A comparative analysis of the complete chloroplast genomes of three Chrysanthemum boreale strains
Source: PeerJ. 2020 Jul 3;8:e9448. doi: 10.7717/peerj.9448 (PMC7337036; doi:10.7717/peerj.9448)

(A)

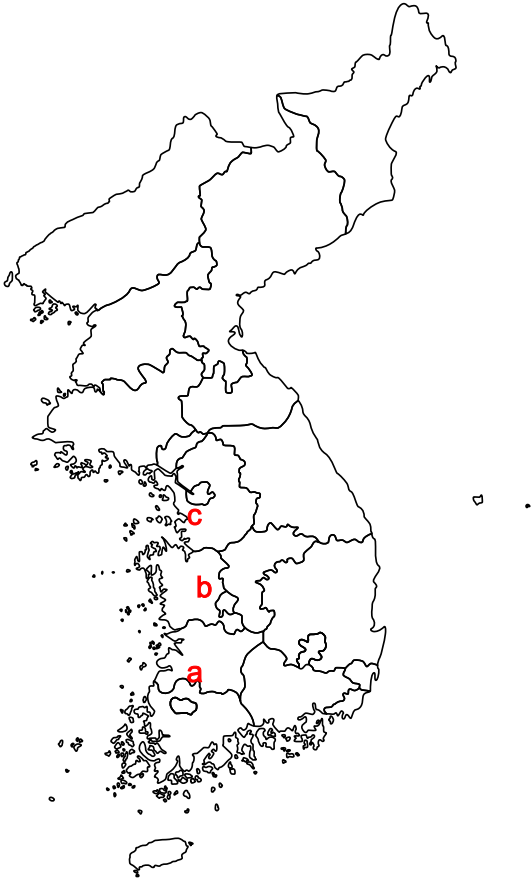

(B)

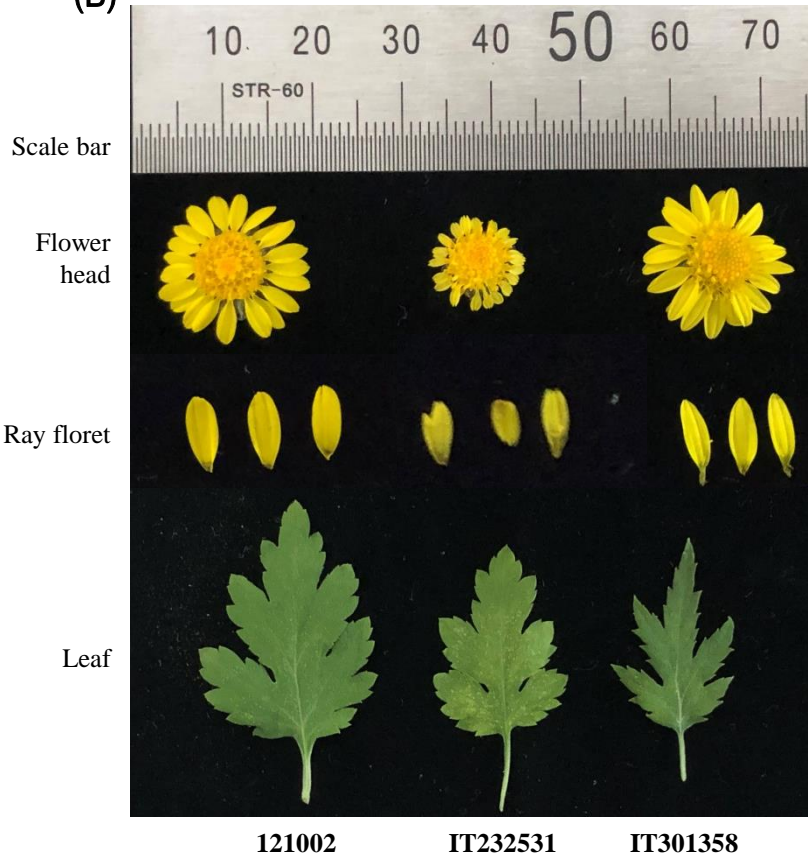

Supplement: Supplemental Information 1 — (A) The collection areas are marked as “a” for 121002, “b” for IT232531 and “c” for IT301358. (B) The morphology of flower head, ray floret and leaf. The ruler scale is in mm. [file peerj-08-9448-s001.pdf]

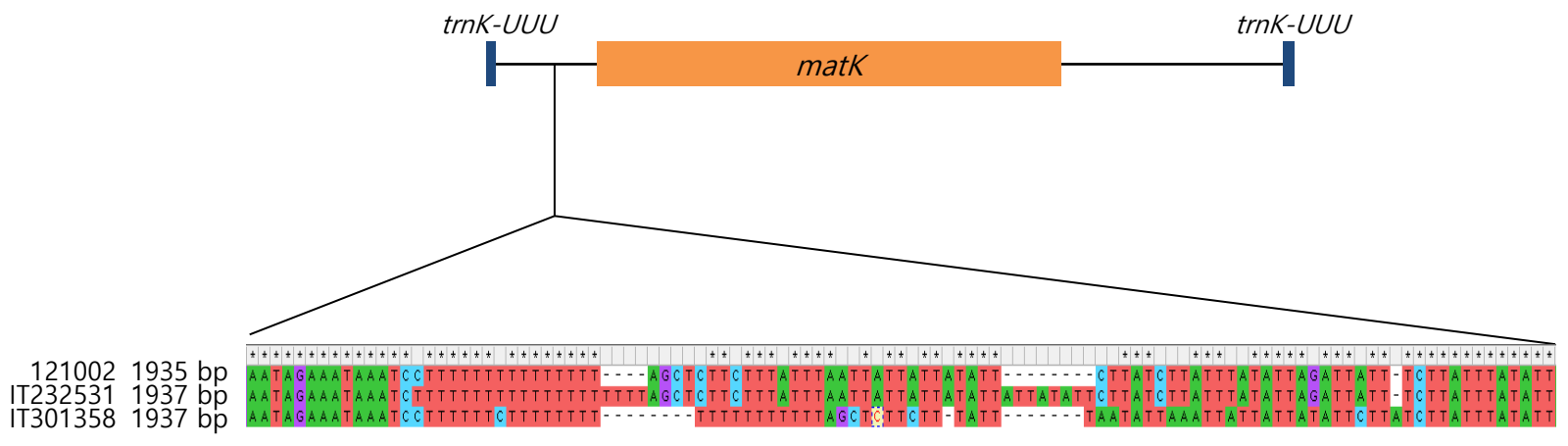

Supplement: Supplemental Information 3 — Only the most divergent regions are shown. The number in bp on the left indicates the position of nucleotide in the complete chloroplast genome. [file peerj-08-9448-s003.pdf]

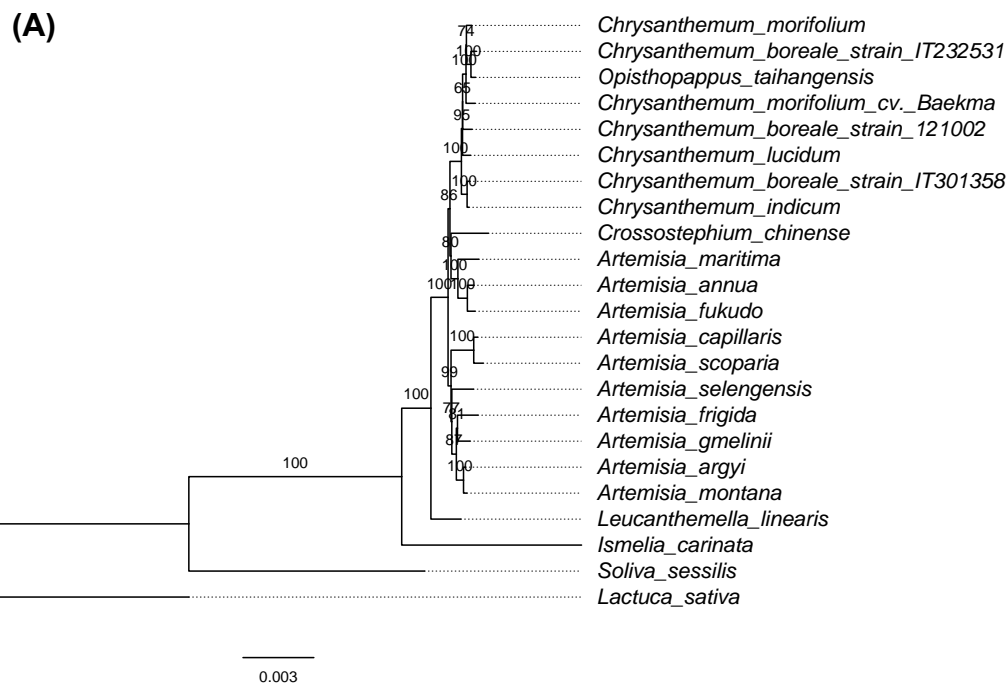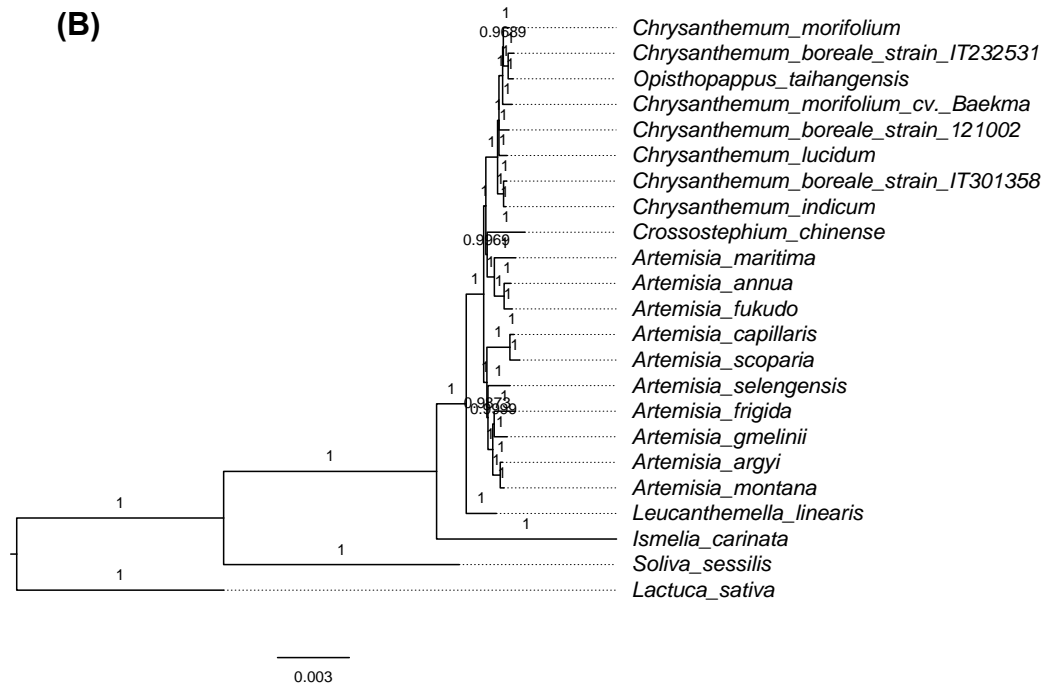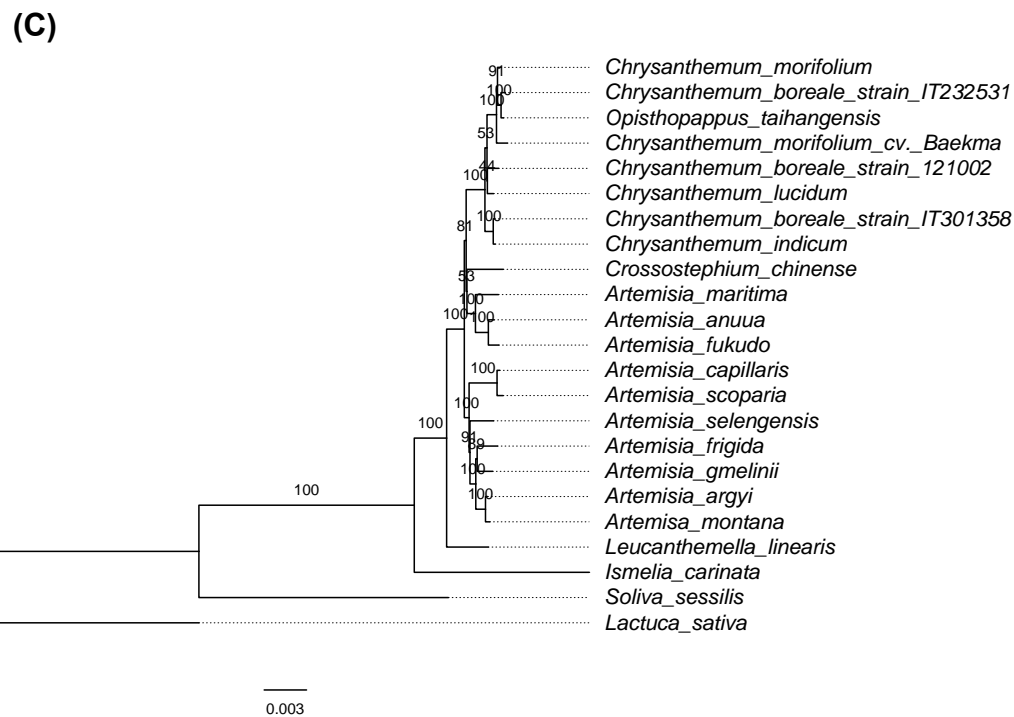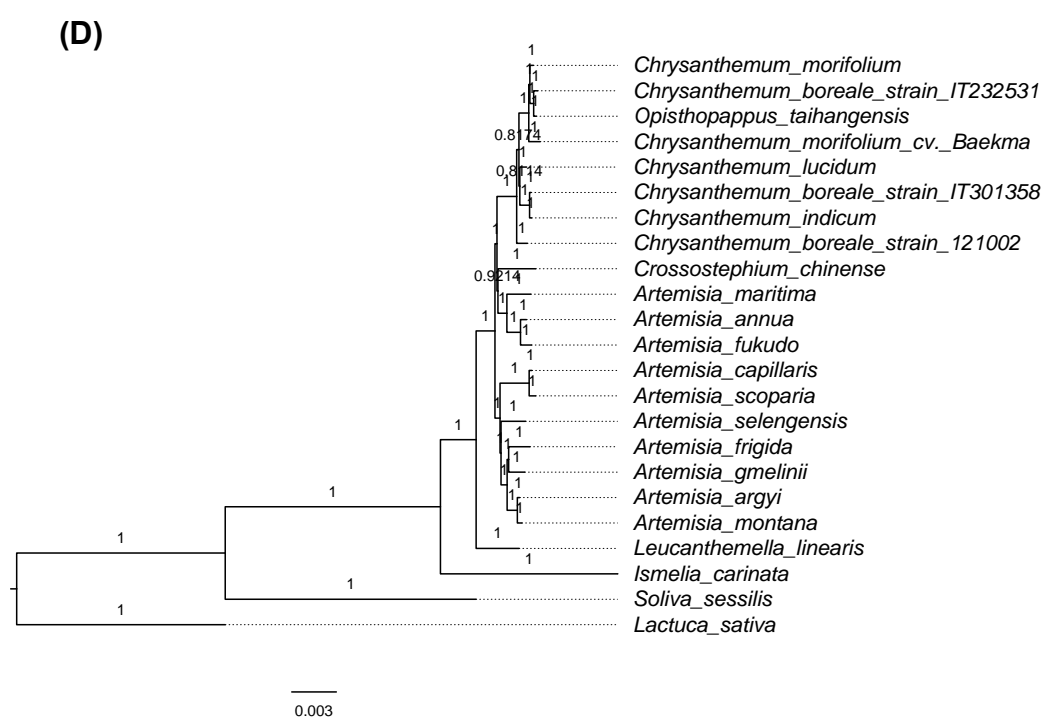

Supplement: Supplemental Information 4 — (A) ML tree based on the sequences of 77 shared protein-coding genes. (B) BI tree based on the sequences of 77 shared protein-coding genes. (C) ML tree based on the complete chloroplast genomes. (D) BI tree based on the complete chloroplast genomes. Numbers above the branches indicate bootstrap support values in ML trees and BI posterior probability in BI trees. The scale bars indicate the number of nucleotide substitutions per site. [file peerj-08-9448-s004.pdf]

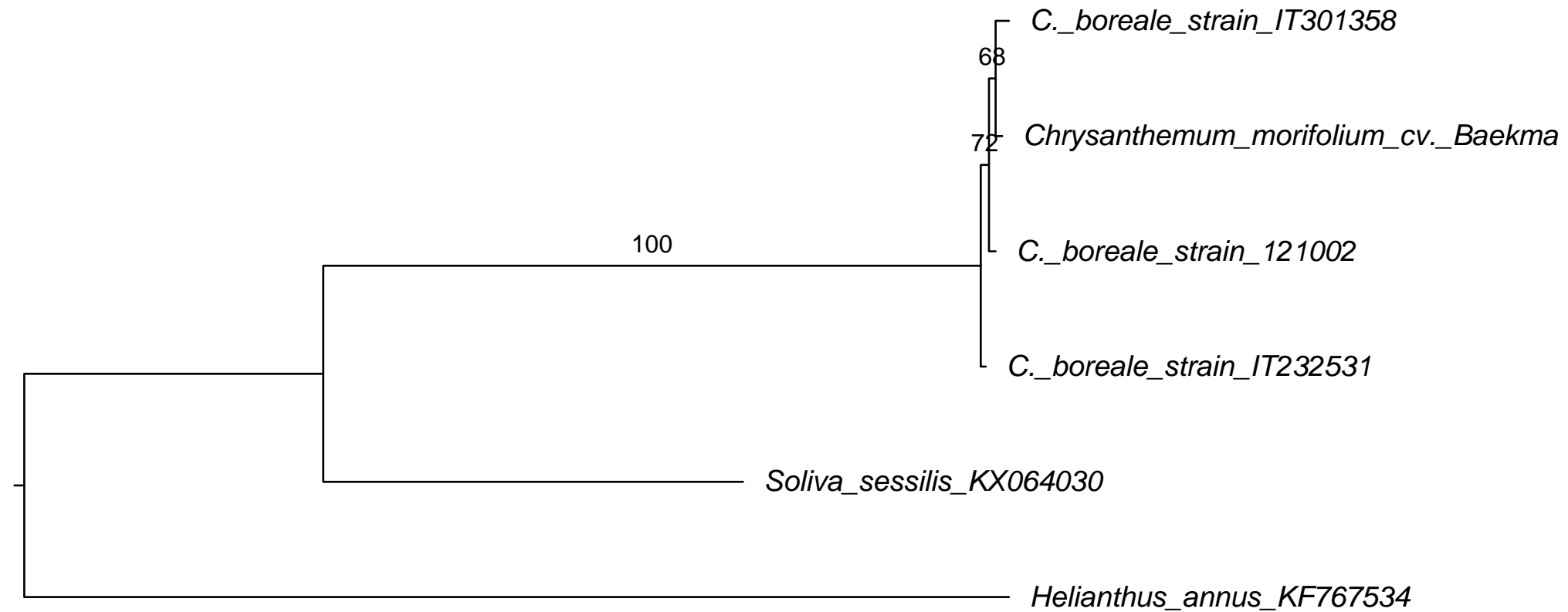

Supplement: Supplemental Information 5 — Numbers above the branches indicate bootstrap support values. The scale bars indicate the number of nucleotide substitutions per site. [file peerj-08-9448-s005.pdf]
