# Supplementary material for: A comparative analysis of the complete chloroplast genomes of three Chrysanthemum boreale strains
Source: PeerJ. 2020 Jul 3;8:e9448. doi: 10.7717/peerj.9448 (PMC7337036; doi:10.7717/peerj.9448)
Supplement: Supplemental Information 2 — Only the inconsistent regions between two assembly processes are shown. The number in bp on the left indicates the position of first nucleotide displayed in the coding sequences of ycf1. [file peerj-08-9448-s002.pdf]

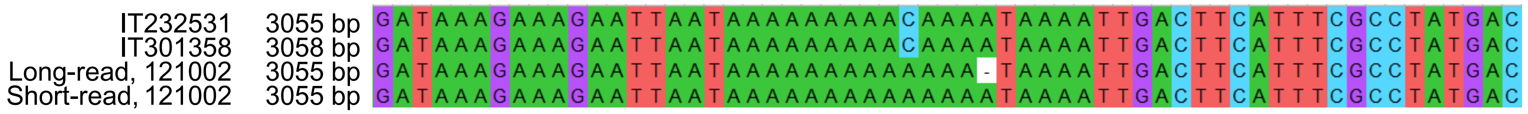

IT232531 3055 bp GATAAAGAAAGAATTAATAAAAAAAAACAAAATAAAATTGACTTCATTTCGCCTATGAC
IT301358 3058 bp GATAAAGAAAGAATTAATAAAAAAAAACAAAATAAAATTGACTTCATTTCGCCTATGAC
Long-read, 121002 3055 bp GATAAAGAAAGAATTAATAAAAAAAAAAAAA-TAAAATTGACTTCATTTCGCCTATGAC
Short-read, 121002 3055 bp GATAAAGAAAGAATTAATAAAAAAAAAAAAAATAAAATTGACTTCATTTCGCCTATGAC
